# Supplementary material for: Are carbon emissions trading and green financial instruments synergistic? -Comprehensive quantitative research based on content analysis
Source: PLoS One. 2024 Mar 7;19(3):e0298601. doi: 10.1371/journal.pone.0298601 (PMC10919601; doi:10.1371/journal.pone.0298601)
Supplement: S1 Data — (ZIP) [file pone.0298601.s001.zip › Data/地级市Z_F工作报告.zip!/╕╩╦α/░╫╥°╩╨/└┤╫╘╣1⁄2╓┌║┼í╛┬φ┐╦╩2╛▌═°í┐_2018.docx]

各位代表：

现在，我代表市人民政府向大会作工作报告，请予审议，并请政协委员和列席人员提出意见。

2017年工作回顾

刚刚过去的一年，面对复杂多变的困难挑战和多年少有的严峻经济形势，在省委、省政府和市委的正确领导下，我们认真贯彻落实党的十八大、十九大精神，以习近平新时代中国特色社会主义思想为指导，全面落实习近平总书记视察甘肃重要讲话和“八个着力”重要指示精神，保持发展定力，聚力攻坚克难，着力推进稳增长、调结构、促改革、惠民生各项工作，全市经济社会发展取得新进展新成效。预计全年完成一般公共预算收入29.94亿元，同比增长4.27%，其中税收收入19.84亿元，增长6.1%;城镇居民人均可支配收入27389元，增长8.2%;农村居民人均可支配收入8309元，增长9%；城镇新增就业5.3万人，完成全年目标任务的106%，比省定目标高1.2万人；地区生产总值445亿元，增长1%;规模以上工业增加值109亿元，增长1.7%；社会消费品零售总额206亿元，增长8%。同时，受民间投资、工业投资、基础设施投资下降和重大项目支撑不足等影响,加上环保政策趋紧、统计政策调整等因素，全市固定资产投资呈负增长，降幅与全省基本持平。在工作中我们始终坚持实事求是，既立足当前集中解决突出问题，又着眼长远谋划推出务实管用举措，更加注重转型升级，更加注重创新驱动，更加注重守住民生底线，经济结构不断优化，质量效益稳步提升，人民群众的获得感持续增强，全市经济社会发展总体平稳健康。

一年来，我们主要做了以下工作：

一、立足补齐短板，脱贫攻坚取得实效。坚持把脱贫攻坚作为全面建成小康社会的底线性任务和标志性任务，紧紧围绕“两不愁、三保障”目标，以深度贫困地区为重点，着力推动脱贫攻坚向纵深发展。强化责任落实，31名市级领导干部、176个市直帮扶单位及驻村帮扶工作队全部调整到37个贫困乡镇、230个贫困村，实现干部帮扶全覆盖。加大资金投入，落实财政专项扶贫资金5.2亿元，整合涉农资金40.95亿元。改善基础条件，新修农村道路491公里；实施农村安全饮水巩固提升工程8处，受益人口27万人；行政村宽带网络实现全覆盖;建成易地扶贫搬迁住宅4945套，搬迁群众1.63万人；改扩建义务教育薄弱学校校舍19.22万平方米，建成行政村幼儿园30所，为34.04万名学生发放助学贷款、助学金5.03亿元；农村贫困人口门诊和住院费用报销比例提高5个百分点，精准扶贫人口大病保险起付线降至3000元，完成26.57万名贫困人口免费体检。发展富民产业，在贫困村种植特色农作物79.17万亩，新建规模养殖场13个，发展农民专业合作社90个，贫困县农产品加工业总产值达到42.57亿元，增长9%；建成电商精准扶贫村级服务站203个，农产品交易额达到2.8亿元，增长47%；转移农村富余劳动力28.13万人，创劳务收入51.8亿元。经过全市上下的共同努力，贫困人口由2016年底的19.87万人减少到14.65万人，贫困发生率下降到10.86%，下降3.87个百分点，圆满完成年度脱贫目标任务。

二、强化统筹调度，经济运行保持平稳。针对年初经济低迷、投资乏力、增速下滑的困局，直面问题找差距，坚持精准施策，从紧迫任务和关键环节入手，强力推进“三重”“三一”工作方案等一系列稳增长政策措施的落实。深入开展“企业服务年”活动，积极支持企业拓宽融资渠道，白银公司成功上市，区域性股权交易中心新增挂牌企业31家，直接融资86.76亿元。1-11月，全市规上企业实现主营业收入802.33亿元，增长10.7%，白银公司、银光公司、稀土公司等骨干企业效益不断好转。扎实开展项目建设“百日攻坚挂图作战”行动，强化项目落地实施和调度管理，71个市列重点项目完成投资152亿元，占固定资产投资的49.8%;争取专项资金73亿元，增长2.3%；签约招商引资项目94个，签约资金216.36亿元。经过不懈努力，广大群众期盼已久的中卫至兰州客运专线、国道341线白银至中川一级公路等重大项目开工建设。国道247线靖远至会宁段改扩建工程建成通车，国道338线景泰至年家井段二级公路完成路基工程，省道217线白银至景泰一级公路半幅通车，黄河白银段防洪工程和城市地下综合管廊试点项目进入收尾阶段。

三、注重提质增效，工业结构不断优化。一手抓传统产业改造提升，一手抓战略性新兴产业培育，制定了推进制造业与互联网融合发展的实施意见，出台了扶持发展壮大有色金属等14个传统产业和新材料、生物医药两个新兴产业的具体措施，不断延伸有色金属及稀土新材料、化工、能源、装备制造等产业链。昌元化工年产10万吨重铬酸钠一期、中瑞铝业一期、华鹭铝业“出城入园”碳素项目建成投产。我市老工业基地改造和传统产业转型升级工作，获国务院通报表扬。郝氏碳纤维循环应用产业示范园、银西生物医药园等战略性新兴产业项目加快实施，银光公司入选全省第四批战略性新兴产业骨干企业。注重发展循环经济，投资39.85亿元，完成高新区循环化改造项目26个，我市被列为中德低碳生态示范试点城市。白银工业集中区“一区六园”完成基础设施投资30.46亿元，新入驻企业47家，吸引投资53.52亿元，园区平台支撑作用逐步增强。

四、推进特色发展，现代农业持续壮大。加快推进农业供给侧结构性改革，优化农业结构，不断提高农业经济效益。全面落实粮食安全责任制，粮食总产量达到83.82万吨，增长5.4%。持续提升瓜菜、草畜、林果、中药材等特色优势产业，新增蔬菜3.35万亩，累计达到38.66万亩；新改扩建标准化规模养殖场50个，肉蛋奶产量达到18.47万吨；新发展苹果、大枣、枸杞等林果4.87万亩，累计达到57.93万亩；新增道地中药材1.9万亩，累计达到17.82万亩。加快推进农业产业化，新增农业龙头企业23家、农民专业合作社350家、家庭农场165家，农产品加工转化率提高到54%，比上年提高1个百分点；农村“三变”改革试点工作正式启动，农村土地确权登记颁证工作基本完成，土地流转新增10.19万亩，流转率达到33.7%。加强农产品品牌建设，新认证国家地理标志保护产品3个，“三品一标”农产品认证面积达到242万亩，占农作物总面积的52%；获得2017年中国绿色食品博览会金奖4个、首届甘肃农业博览会金奖1个，农产品品牌的知名度和影响力不断提高。

五、强力推动创城，生态家园更加美好。把创建全国文明城市牢牢抓在手上，强力推进基础设施建设和环境整治，城乡面貌明显改善，天更蓝了、水更清了、地更净了，城市的“精气神”更足了，赢得了群众点赞。一年来，市区改造提升道路23条、背街小巷38条、公园广场8个，成功打造滨河东路、长安路等多条标准化路段；亮化各类建筑530栋，新设标识牌1200个，清理各类广告牌1.7万块；新增绿化面积150多万平方米，市区绿化覆盖率达到34.68%。加大城乡环境整治，清理河道沟渠、村组道路7800公里，清运垃圾62万吨。完成各类营造林38.59万亩，全市森林覆盖率达到13.53%。新建省级“千村美丽”示范村13个、市县级美丽乡村示范村64个、环境整洁村138个，打造了“花村·顾家善”等一批展示白银美丽乡村建设成果的亮丽名片。我市被评为第五届全国未成年人思想道德建设先进城市，3个村镇、4个单位、1所学校分别被评为全国文明村镇、文明单位和文明校园。认真落实河长制，324条（段）河道设立河长，巡河工作有序推进。落实耕地保护责任，实施土地整治项目35个，治理水土流失面积140平方公里。以祁连山生态环境破坏问题为警示，积极整改中央环保督察反馈问题，东大沟流域重金属污染整治及生态修复、历史遗留含铬土壤污染治理、黄家洼区域生态环境恢复治理等工作稳步推进，完成燃煤电厂超低排放改造和稀土冶炼尾气治理等项目10个。严格管理烟花爆竹燃放，市区燃放现象基本消除。整治燃煤小锅炉480台，淘汰老旧机动车、“黄标车”9675辆。全年市区空气质量优良天数达到304天，同比增加5天，市区PM10平均浓度同比下降3.4%，PM2.5平均浓度下降13.2%，圆满完成大气污染防治年度目标任务。

六、突出着力重点，第三产业稳步发展。加快文化旅游融合发展，积极开展“全域旅游示范区”创建，建成运营“花映盛世”景观带、黄河石林国际滑雪场、龙湾村民俗改造、东湾薰衣草庄园等一批文化旅游景点，成功举办黄河风情文化旅游节、水川荷花美食文化节、靖远枸杞爱心采摘节、第十七届PHE国际青少年儿童书画大会等一系列文化旅游节会。全年接待游客突破1000万人次，实现旅游综合收入63亿元；培育发展文化企业33家，文化产业增加值达到7.59亿元，增长12%。加快商贸物流基础建设，中进装饰建材城投入运营，甘肃邦农农产品批发市场被列入省级八大农贸市场之一，银东工业物流园、天奇仓储物流园、靖远西北果蔬冷链物流中心等项目加紧建设，鼎丰物流海关监管场所获批，建成县乡农贸市场9个。推进“一带一路”物流集散基地建设，首列白银至阿拉木图中欧班列开通运行。狠抓电子商务，国家电子商务进农村综合示范县项目顺利推进，电商企业、网店分别达到216家、4430家，实现交易额6.7亿元，增长40%。

七、着眼增强动能，改革创新深入推进。深化供给侧结构性改革，扎实推进“三去一降一补”，淘汰“地条钢”、水泥等落后和过剩产能192万吨，商品房去库存64.57万平方米，刘川工业园被列为全省增量负荷供用电体制改革试点，规上工业企业每百元主营业务收入成本下降1.15元。取消调整下放行政审批事项88项，下移城市管理重心，将市级15项城市管理行政审批事项下放到白银区，将黄河石林风景旅游区管理权限下放到景泰县。不断深化商事制度改革，新发展各类市场主体17486户，增长18.66%。供销合作社改革稳步推进。加快兰白科技创新改革试验区建设，省级创新型试点城市通过评估验收，“六个一百”技术创新工程深入实施，争取技术创新驱动基金、孵化器专项基金2.86亿元，转化重大科技成果21项，新增高新技术企业21家，建设创新平台23个；科技企业孵化器新入孵企业24家，出孵企业8家。突出抓好高端人才引进、“人才归根”工程和首席专家队伍建设，建立院士工作站7个，柔性引进院士7名。新增发明专利申请136件、授权39件，全市有效发明专利达到313件，居全省第二位。成功举办第二届创新创业大赛，发放创业担保贷款1.5亿元，创建省级创业孵化基地8个。

八、坚持共建共享，民生福祉显著增进。全市财政用于民生领域支出139.4亿元，占一般公共预算支出的87%。省市确定的20件惠民实事基本完成。城乡低保标准分别提高8%、22.6%，城乡居民基本医保补助标准人均达到450元。建成棚改安置房2390套，改造农村危房1万户。全市义务教育巩固率达到99.7%，学前三年、高中阶段毛入学率分别达到92.3%、94.3%，会宁县通过义务教育均衡发展评估验收。在全省率先推行公立医院药品采购“两票制”，市第一人民医院住院部综合大楼投入使用，市第三人民医院后勤保障楼、市妇幼保健院业务楼、市疾控中心实验楼基本建成，城乡居民大病保险补偿标准提高到人均55元，新农合参合率达到97.09%。积极创建国家公共文化服务体系示范区，建成街道社区综合文化服务中心55个、“乡村舞台”83个，“靖远民歌”、“景泰滚灯”等入选第四批省级非物质文化遗产代表性项目目录，火焰山国家矿山公园免费向游客开放。成功举办第22届全国大学生乒乓球锦标赛、全国乒乓球少儿杯赛总决赛和市第四届运动会等体育赛事，建成“一村一场”农民体育惠民广场100个。深入开展双拥共建，退役士兵安置和权益保障政策全面落实。扎实推进“七五”普法，建成村（社区）司法行政工作室493个，矛盾纠纷化解率和信访积案办结率分别达到98.9%和96%。深化拓展平安白银建设，依法打击违法犯罪活动，刑事案件发案同比下降23.2%。民族团结、宗教和顺的局面进一步巩固，靖远红嘴村被评为2017年全国民族团结进步创建示范单位。强化产品质量、食品药品安全和物价监管，市场秩序平稳有序。牢固树立安全发展理念，大力推进安全标准化建设，加强重点行业和重点领域隐患排查治理，严厉打击非法生产经营建设行为，安全生产形势总体平稳。

过去的一年，我们坚持把全面从严治党要求落实到政府工作全过程、全领域，牢固树立“四个意识”，扎实推进“两学一做”学习教育常态化制度化，深入开展“三纠三促”、村级组织集中整治、基层社会治安综合治理专项行动，作风建设持续深化。修订完善市政府工作规则，认真落实市政府常务会议会前学法制度，严格执行重大行政决策规则，科学决策、民主决策和依法决策水平不断提高。自觉接受人大法律监督、政协民主监督，90件人大代表建议、175件政协提案全部办复。加强审计监督，纠正违规资金3.3亿元。规范公共资源交易，市级政府采购节约资金1.78亿元。强化行政监察，立案查处违纪案件504件，党纪政纪处分660人。与此同时，外事侨务、防震减灾、妇女儿童、红十字会、残疾人事业、住房公积金、地方志、统计、档案、气象、人防、老龄等工作也取得了新成绩。

各位代表！一年来的成绩来之不易，这是省委、省政府亲切关怀的结果，是市委坚强领导的结果，更是全市人民奋力拼搏的结果。在此，我代表市人民政府，向全市各族人民，向各民主党派、工商联、无党派人士、人民团体和社会各界人士，向驻银部队、武警官兵、公安干警和中央、省驻银单位，向所有关心、支持白银改革发展的各界朋友，致以崇高的敬意和衷心的感谢！

同时，我们也清醒地认识到，我市发展不平衡不充分的问题仍然很突出，政府工作中还存在许多不足和问题，也面临不少困难和挑战。一是脱贫攻坚任务繁重，全市还有贫困人口14.4万人，特别是会宁、靖远两县被列为深度贫困县，全市有3个乡镇及179个村被列为深度贫困乡镇和深度贫困村，我们攻克难中之难、坚中之坚必须付出更加艰辛的努力。二是生态环境脆弱仍是基本市情，生态环保形势还非常严峻，实施国土绿化和生态修复任务艰巨，构建绿色生态产业体系显得十分紧迫。三是经济持续稳定增长的动力不足，受多重因素影响，生产总值、固定资产投资、规模以上工业增加值等经济指标增速下滑的势头尚未扭转；制约经济社会发展的短板依然突出，实体经济实力还有待提升，民营经济发展缓慢，产业结构性矛盾突出，新旧动能转换任务艰巨；农田水利、农村道路、对外通道、通讯信息、公共服务等领域基础建设欠账较大。四是县域经济发展滞后，主导产业发展层次不高，支撑县域经济发展的产业化大项目、高附加值项目缺乏，园区配套设施不完善，县区财政自给率普遍偏低，三个县均不超过11%。五是科技创新引领作用发挥不够充分，科技成果转移转化的通道机制还不完善，企业创新主体地位还不凸显，兰白科技创新改革试验区示范性、引领性项目不多，科技资源的聚合度不强。六是政府职能转变还不到位，“放管服”改革的成效与企业和群众的满意度还有差距，营商环境还不够好；一些干部主动担当作为不够，还存在本领恐慌、能力不足、工作懈怠等问题。对于工作中存在的这些困难和问题，我们要勇于面对，认真分析研究，切实加以解决。

2018年主要目标和重点工作

今年是贯彻党的十九大精神的开局之年，是改革开放40周年，也是决胜全面建成小康社会、实施“十三五”规划承上启下的关键一年。机遇和挑战并存，机遇大于挑战。从外部环境看，主要发达国家和新兴市场国家经济继续好转，全球经济有望保持温和复苏势头；我国经济运行呈现出稳中向好的良好态势，发展的包容性和获得感明显增强；我省“三重”“三一”等一揽子稳增长配套跟进政策效应逐步释放，全省经济正努力走出短期阵痛期，总体向好迹象愈加明显。从政策机遇看，党的十九大作出统筹推进“五大建设”、坚定实施“七大战略”、坚决打好“三大攻坚战”等重大部署，中央经济工作会议提出高质量发展要求和建设现代化经济体系的重点任务，为我们做好工作指明了前进方向、提供了根本遵循；省委省政府贯彻落实习近平总书记视察甘肃“八个着力”重要指示精神，作出的加快推进深度贫困地区脱贫攻坚、老工业基地振兴、兰白科技创新改革试验区建设、兰白都市圈为核心的中部城市群建设等部署，为我市发展提供了政策支持和强大动力。从发展基础看，经过长期努力，我市在转型升级、“一区六园”建设、基础设施建设等方面打下了良好基础，正在形成新的比较优势，有望进入经济规模质量同步提升、新型工业化稳步推进、新业态新动力蓄势发力的重要阶段，处于大有可为的战略机遇期。只要我们正确把握大局，遵循发展规律，坚定发展信心，凝聚共识合力，就一定能够战胜前进道路上的艰难险阻，推动经济社会发展再上新台阶，更好地满足全市人民日益增长的美好生活需要。

政府工作总体要求是：全面贯彻落实党的十九大精神，以习近平新时代中国特色社会主义思想为指导，深入落实习近平总书记视察甘肃重要讲话和“八个着力”重要指示精神，坚持稳中求进工作总基调，积极践行新发展理念，紧扣解决不平衡不充分的发展问题，以供给侧结构性改革为主线，统筹推进稳增长、促改革、调结构、惠民生、防风险各项工作，大力实施工业强市战略和乡村振兴战略，更加突出创新驱动，着力打好防范化解重大风险、精准脱贫、污染防治攻坚战，不断增进民生福祉，奋力开创全市经济社会持续健康发展新局面。

经济发展主要预期目标是：生产总值增长5％，规模以上工业增加值增长3%，固定资产投资增长7％，社会消费品零售总额增长8％，一般公共预算收入增长6％，城镇居民人均可支配收入增长7％，农村居民人均可支配收入增长7.5％，新增城镇就业4.2万人以上，居民消费价格总水平涨幅控制在3％以内。

为实现上述目标，我们将重点做好以下六个方面的工作：

一、抓好精准脱贫，下足绣花功夫，切实补齐全面小康短板。把脱贫攻坚作为政治任务、头等大事和第一民生工程来抓，聚焦深度贫困乡村和建档立卡贫困户，坚持“两不愁、三保障”脱贫标准，实行“七个一批”清单管理，精准施策，确保5.03万人稳定脱贫。

加快补齐脱贫短板。加强政策研究和对接争取，确保国家对深度贫困地区土地、基础设施、教育、就业、兜底保障等一系列支持政策在我市落地见效。牢牢抓住省上1000亿元农村人居环境综合治理工程专项贷款机遇，实施贫困村整体提升工程，加快补齐贫困乡村基础设施和基本公共服务短板。硬化农村道路2269公里，实现深度贫困县30户以上、其他县区50户以上具备条件的自然村全部通硬化路。抓好兴电灌区渠道维修、正路提灌工程、戈壁农业调水等水利扶贫项目，着力改善贫困乡村水利基础设施。实施农村饮水安全巩固提升工程9处，受益人口35万人。升级改造农村电网209公里。改造农村危房9046户。加快65个易地扶贫搬迁安置点建设，确保7993户、3.21万人顺利搬迁。加强教育扶贫，加大贫困家庭学生救助力度，新改建农村薄弱学校校舍12万平方米。持续开展健康扶贫“三个一批”行动计划，有效解决因病致贫、因病返贫问题。强化社保兜底，实现农村低保标准和国家扶贫标准“两线合一”。

切实增强脱贫内生动力。发挥产业扶贫关键作用，主动作为，积极争取，用足用好省上1000亿元特色产业发展工程贷款和500亿元产业发展投资基金扶持政策，重点支持富民产业培育。积极对接省上乡村旅游精准扶贫工程，集中扶持一批贫困村发展乡村旅游。创新贫困地区种养大户、农民专业合作社、龙头企业等经营主体与贫困户的合作共赢机制，增强贫困群众持续增收能力。改进农业实用技术、务工技能培训方式方法，提高劳务输转组织化程度，完成各类职业技能培训2.4万人，转移农村富余劳动力28万人。坚持扶贫与扶智、扶志相结合，完善生产奖补、劳务补助、以工代赈等机制，教育和引导广大群众通过辛勤劳动实现脱贫致富。

不断完善脱贫攻坚机制。围绕省上贫困人口脱贫11项指标、贫困村退出20项指标和贫困县摘帽7项指标，科学编制深度贫困乡村脱贫攻坚方案。严格落实增列专项扶贫预算政策，加大涉农资金项目整合力度，新增脱贫攻坚资金和项目主要用于深度贫困乡村，惠民项目向深度贫困乡村倾斜。加强与天津对口帮扶单位的对接交流，协作开展“携手奔小康行动”，在资金项目、学生就业、劳务输转、学习培训等方面争取更多支持。深化“百企帮百村”行动，鼓励各类社会组织开展到村到户精准扶贫。完善脱贫攻坚监督考核机制，加强扶贫领域资金监管，严肃处理数字脱贫、弄虚作假以及贪污挪用扶贫资金等问题，强化扶贫领域作风建设，做到脱真贫、真脱贫。

着力壮大县域经济实力。把发展县域经济作为打赢脱贫攻坚战、决胜全面小康的关键举措，加大对县域经济发展的规划和指导，编制县域经济发展规划、特色产业规划和城乡基础设施规划，提升市县联动发展、县域自主发展水平。运用好贫困县IPO绿色通道政策洼地，积极引入和培育符合上市、挂牌条件的优质企业，提升贫困县经济实力。采取鼓励金融机构增加信贷投放、探索设立市县产业投资发展基金等方式，支持县区争取项目资金、争创各类试点示范、引进战略投资者，培育一批特色鲜明、带动力强、发展潜力大的特色产业和龙头企业，促进人口和产业聚集，激发县域经济内生动力。

二、注重绿色发展，改善生态环境，加快推进美丽白银建设。牢固树立社会主义生态文明观，着眼打赢污染防治攻坚战，重点是打赢蓝天保卫战，持之以恒推进生态环境保护和综合治理，建设绿色生态美好家园。

加快生态建设步伐。推进国家级气候适应型城市建设试点工作，切实提高城市适应气候变化能力和水平。加大国家水土保持重点工程建设力度，新修梯田5万亩，完成水土保持综合治理140平方公里。总结推广景泰县“挖塘降水、抬土造田、渔农并重、治理盐碱”的做法，加快推进土地盐碱化治理。全面推行“河长制”，严守水资源管理“三条红线”，推进水生态环境持续改善。加强沙化地区防沙治沙，建设防沙治沙综合示范区。全面启动国土绿化行动，推进退耕还林还草、天然林保护、三北防护林等重点生态工程建设，完成营造林面积32万亩。

加大环境综合治理。实行联动、销号、预警、公告四项整改工作机制，认真抓好中央环保督察反馈问题整改。加强大气污染防治，强化“六张清单”管理，严格落实“六个百分之百”城市扬尘管控措施，推进热电联产和集中供热，完成燃煤小锅炉治理，淘汰黄标车和老旧机动车1万辆以上，确保市区空气质量优良天数达到300天以上。深入实施“水十条”，推进水污染防治，加强城镇污水处理设施建设，提高污水处理率；强化饮用水水源环境保护，推进祖厉河流域污染整治，县级及以上城市集中式饮用水源水质优良比例达到100%。全面实施“土十条”，抓好土壤污染防治，继续推进白银区东大沟河道重金属污染、历史遗留铬污染土壤治理工程建设，启动实施银光公司土壤硝基化合物污染治理工程。整合环境执法力量，强化生态环境执法监管，对环境违法行为发现一起、查处一起。

促进循环绿色发展。加快推进循环经济示范城市建设，开展重点用能单位“百千万”行动，加大节能减排降碳力度，确保完成年度节能降耗目标任务。提高农业废弃物资源化利用，农作物秸秆资源综合利用率、废旧农膜回收率、规模化畜禽养殖废弃物利用率分别达到82%、80%和85%以上。严格执行建筑节能标准，推广装配式建筑，新建建筑执行节能强制性标准比例达到97%以上。持续开展节约型机关、绿色家庭、绿色学校、绿色社区创建和绿色出行行动，抓好4个省级低碳社区试点建设，有效控制城乡建设和居民生活领域温室气体排放。启动实施流通领域“限塑”行动，坚决遏制“白色污染”。

加强城市建设管理。持续推进全国文明城市、国家卫生城市和国家园林城市创建工作，不断提升城市建设管理水平。发挥规划引领作用，编制完成城市总规近期建设、沿黄田园小城镇带等规划，启动“多规合一”编制工作。认真做好城市设计，提升城市风貌及建筑风格整体水平。加快中心城区基础设施建设，完成城市地下综合管廊试点项目收尾和北京路、银山路等道路提升改造，抓好公园广场、城市出入口等重要区域景观建设和绿化管护。用足用好棚户区改造政策，启动新的棚改攻坚计划，完成棚户区改造6128户（套）。加大城区停车位规划建设力度，积极解决市民停车难问题。提升县城建设水平，规划实施靖远靖乐渠环境治理、景泰三水一园一河生态修复等工程。加快智慧城市建设，推进城市管理服务平台与应急指挥、城区路灯监控、民情服务热线等系统的对接、推广、应用工作，提高城市管理网格化、信息化、精细化水平。加大对违法建设、马路市场以及乱停车、乱占道等行为的整治力度，完善市政设施、园林绿化、环卫保洁等长效机制，为广大市民营造整洁有序的生活环境。

三、突出项目建设，扩大有效投资，全力推动经济平稳增长。坚定不移把发展作为第一要务，以项目扩投资，以投资稳增长，持续增强经济发展的支撑力。

强力推进项目建设。全年计划实施亿元以上项目178个，年度完成投资237亿元;实施市列重点项目50个，年度完成投资125亿元。准确把握国家和省上重大战略、产业政策、资金投向和扶持重点，着力在产业转型升级、基础设施建设、社会事业发展、生态环境保护等方面，深入论证谋划一批大项目、好项目。加大前期投入，主动对接争取，力促黄河上游白银段生态环境综合治理、甘肃中部生态移民扶贫开发供水工程、环县至海原至中川铁路、白银至平凉铁路会宁段等重大项目前期工作取得实质性进展。全面落实重点项目领导包抓责任制，紧扣项目申报、资金筹措、用地保障等关键环节，积极协调服务，强化督查调度，开工建设省道35线景泰至会宁高速公路、国道341线辘辘坝至白银段、省道209线郭城驿至巉口二级公路改造提升、靖远县城至刘川工业园快速通道、会宁长征南路拓宽改造等新建项目，全力加快中卫至兰州客运专线、国道341线白银至中川机场一级公路、景泰至中川高速公路、省道217线白银至景泰一级公路、靖远金滩黄河大桥等续建项目进度，确保省道209线平川至九寨沟公路平川段、会宁县城集中供热改造等项目建成投用。

全力做好招商引资。坚持引资引智引技并举，紧盯前沿技术和产业链，瞄准高成长性科技型中小企业，开展产业链招商、点对点招商、园区招商，力争引进一批关联度高、带动力强的龙头型、科技型项目。积极吸引东部、京津冀地区产业转移，建设兰白承接产业转移示范区。引导本土企业与招商引资项目务实合作、借船出海，提升融合度。完善招商引资工作机制，落实各县区、各部门和园区招商引资责任，切实提高招商引资合同履约率、项目开工率和资金到位率，全年签约招商引资项目100个以上，到位资金达到200亿元以上。

着力激发民间投资。落实好鼓励支持民间投资的政策措施，对民间资本可以进入的领域，下大力气打破隐性壁垒，推动全领域、全产业链向民间资本开放。充分发挥政府政策、资金、资源“四两拨千斤”作用，在基础设施和医疗、养老、教育等公共服务领域，规范推行政府和社会资本合作模式，不断充实完善PPP项目库，筛选推介一批前期工作成熟、具有长远盈利预期的PPP项目，力争民间投资占固定资产投资比重达到60%以上。大力发展民营经济，深入实施民营企业“双十百”培育工程。制定出台构建亲清新型政商关系的具体规定，畅通企业表达诉求的途径和渠道，积极主动为企业排忧解难。

四、加快结构调整，推动转型升级，努力实现更高质量发展。围绕建设现代化经济体系和推动高质量发展，大力发展实体经济，实现更有效率、更可持续、更高质量的增长。

深入实施工业强市战略。进一步强化工业主导地位，落实好《中国制造2025白银实施方案》，着力推动产业集群发展。加快改造升级传统产业，促进有色、化工、能源等传统产业延链补环，推动白银公司湿法炼锌渣综合回收及无害化处理、东方钛业资源综合利用等项目开工建设，确保昌元化工10万吨重铬酸钠二期、白银公司铜冶炼技术提升改造、中天化工年产2000吨电子级超纯氟化锂等项目建成投产；支持靖煤公司探索发展煤制天然气、煤制油、煤制乙醇等新型煤化工项目和清洁能源利用项目。培育壮大战略性新兴产业，重点推进郝氏碳纤维循环应用产业示范园、皓天原料药、康奇医药年产1万吨医药中间体、贝达化工中间体及环保新材料、新乐雅分布式光伏发电等项目建设，谋划推动凹凸棒石产业技术研发及开发利用项目，培育一批具有核心竞争力的高新技术企业，争取规上战新产业增加值增长8%。加快军民融合创新示范区建设，支持银光公司优化含能材料生产工艺，并依托光气化产品延伸加氢精细化工产业链；支持稀土公司推动稀土和凹晶材料军民市场开发，切实将资源、技术优势转化为经济优势。积极帮助企业脱困发展，加大企业帮扶力度，解决企业在市场、资金、要素等方面的难题，促使企业复产、达产、扩产。开展规上企业培育计划，抓好“个转企”、“小升规”工作，新增规上工业企业15户。

大力实施乡村振兴战略。全面贯彻落实中央农村工作会议精神，把实施乡村振兴战略作为做好“三农”工作的总抓手，按照产业兴旺、生态宜居、乡风文明、治理有效、生活富裕的要求，科学制定乡村振兴战略规划，加快农业农村现代化步伐。积极推进农业供给侧结构性改革，优化农业产业结构，在保障粮食安全的基础上，加快发展以设施蔬菜、高原夏菜为主的瓜菜产业和以苹果、梨、大枣、枸杞为主的果品产业，因地制宜扩大黄芪、党参、甘草等道地中药材和甜叶菊种植规模，新增蔬菜种植2万亩、林果10万亩、中药材1万亩。稳定发展以牛、羊、黑毛驴、奶骆驼等为主的草食畜牧业，新改扩建标准化规模养殖场（小区）40个。构建多元产业体系，抓好农产品精深加工，加快推进鑫昊乳制品加工、菁润大枣精深加工等项目建设，新增农业龙头企业15家，农产品加工转化率达到55%。积极对接省上“千合农庄计划”项目，以“公司+合作社+农户”的模式，实现“农业+民宿+园艺+旅游+电商”的集约化发展。大力发展现代农业产业园、科技园、创业园和田园综合体，加快白银国家农业科技园区建设，抓好白银荔森现代生态农业示范园、鼎鑫靖远现代高效循环农业示范园等项目，推动会宁南川、靖远东湾、平川枣树台等11个田园综合体建设，促进农村一二三产业融合发展。坚持质量兴农、绿色兴农，实施品牌提升计划，健全农产品质量和食品安全标准体系，积极创建国家、省级农业标准化示范基地、畜禽标准化示范场和无公害产地认证，形成规模优势和品牌优势，力争“三品一标”农产品面积占农作物播种面积的52%以上。坚持把全域无垃圾工作作为乡村振兴战略的突破口，扎实开展全域无垃圾三年专项治理行动，全面推进城镇村庄、集贸市场、河道水体、交通沿线等重要节点的环境整治，健全完善垃圾收集、处理和日常保洁等机制，集中打造一批农村环境整治样板村。加强农村公共文化建设，开展移风易俗行动，提升农民精神风貌。统筹推进特色小镇和美丽乡村建设，加快景泰黄河石林、白银水川康养、平川陶瓷等特色小镇建设步伐，建成美丽乡村示范村97个。

着力促进服务业加快发展。坚持以满足需求、促进消费为导向，推动现代服务业比重提高、效益提升。推进文化旅游业融合发展，实现文化产业增加值增长10%以上，旅游综合收入增长25%以上。推进黄河石林大景区、会宁全国红色旅游经典景区、黄河石林国际露营地、黄河假日城湿地风情园、工矿遗址与现代工业观光体验游等项目建设；按照“一水、两路、六湾、八景、十村”规划，实施金沟河综合治理工程，推进白银水川国际慢城旅游度假区建设，打造集旅游观光、健身娱乐、休闲度假为一体的城乡生态长廊，提升文化旅游整体水平。大力发展商贸物流业，实施好白银综合物流园、鼎丰综合保税物流园、会宁物流园、红星美凯龙家居博览中心等项目，培育大中型商贸流通限上企业15家以上，加快建设工业物流集散中心。推动金融创新，加大对“三农”、中小微企业等重点领域信贷投入，协调推进农村信用社改制农村商业银行，引导金融机构增强服务实体经济能力；做好金融风险防控，坚决打击违法违规金融活动。抓好国家电子商务进农村示范县建设，培育壮大本土骨干电商企业，推进实体店网上商城建设，实现线上线下融合发展。突出业态创新、管理创新和服务创新，推动餐饮、家政、健康、养老等服务业便利化、精细化、品质化发展。

加速推动园区提档升级。按照主业突出、特色鲜明、错位发展思路，推动产业向园区集中、企业入园发展，力争“一区六园”新入驻企业50家，完成投资85亿元，产值达到650亿元以上。加快园区供电、供水、道路、污水处理等基础设施建设，重点实施好高新区、银东工业园、正路工业园基础设施建设等项目，全面完成刘川工业园净水厂、平川经济技术开发区和银西生态产业园污水处理厂项目，提高园区承载能力。优化园区管理服务，创新投融资机制和园区建设模式，健全创新创业服务平台，推进项目帮办服务流程优化再造，营造更加优良的园区发展环境。

五、坚持改革创新，扩大开放合作，持续增强发展内生动力。坚持用改革的举措破解难题，用创新的方式驱动发展，用开放的办法拓展空间，为转型发展注入新活力、增添新动力。

着力加快创新型白银建设。认真贯彻落实省上创建兰白国家自主创新示范区要求，用足用活各项支持政策，加大科技创新力度，力争全社会研发投入占生产总值比重提高到1.2%，科技对经济增长的贡献率提高1个百分点。突出企业创新主体地位，支持企业与高校、科研院所开展产学研合作，建立省市级工程技术研究中心3家；帮助企业用好兰白试验区技术创新驱动基金、科技投资基金和创新创业投资基金，加快培育一批标志性、引领性科技示范项目和示范企业，全年培育高新技术企业10家、科技型企业15家。促进科技成果转化应用，加快孵化器和加速器建设，构建“创业苗圃+孵化器+加速器”的全产业链孵化链条，打造一批创客空间、创新创业中心和新兴产业“双创”示范基地，新招孵企业30家以上，实施科技成果转化项目15项，推动形成科技同经济对接、创新成果同产业发展对接、科研项目同现实生产力对接的良好格局。深化科技合作交流，加强与上海张江、天津滨海等自主创新示范区的合作，加快推进兰州大学白银产业技术研究院、兰州理工大学白银新材料研究院建设，引进落地一批重大科技项目、高端研发团队、创新基地与服务平台。加强科技人才队伍建设，实施急需紧缺高层次人才柔性引进计划，加大创新型人才和创新团队引进，推进院士工作站建设，加快培育本土创新型和高技能人才，进一步激发各类人才创新创业活力。

深入推进“三去一降一补”。去产能方面，坚决防止“地条钢”死灰复燃，稳步推进煤炭火电行业化解过剩产能，加快清理僵尸企业，帮助已化解过剩产能企业脱困发展。去库存方面，落实租购并举的住房制度，用好住房公积金、棚户区改造货币化安置等政策，商品房去库存50万平方米。去杠杆方面，有序开展市场化银行债权转股权，提升企业直接融资能力，帮助企业减轻债务负担，有效降低企业杠杆率。降成本方面，进一步细化实化各项降成本措施，加大对中小微企业的扶持力度，降低各类交易成本特别是制度性交易成本。积极争取扩大大用户直购电范围和规模，力争银西产业园“互联网+智慧能源”项目落地实施，降低企业用电成本。补短板方面，主要从制约经济社会发展的重要领域和人民群众迫切需要解决的突出问题入手，着力补齐脱贫攻坚、基础设施、公共服务、产品质量、生态环境等方面的发展短板和制度短板。

加大重点领域改革攻坚力度。不断深化“放管服”改革，从实际出发继续精简市本级行政审批事项，加强培训指导、业务对接和保障，提高县区承接能力；全面推行并联审批、网上审批，实现投资项目在线审批全网通办；紧盯投资管理、安全生产、环境保护等关键领域，创新监管方式，强化事中事后监管，实现“双随机一公开”全覆盖；推行“互联网+政务服务”，推动实体政务大厅与网上服务平台深度融合，进一步提高服务质量和效率。加快推动投融资体制改革，抓好企业投资项目承诺制试点工作，对政府核准的投资项目和负面清单外的企业投资项目，实行“承诺备案制”和“零等待”办理，激发社会投资活力。建立健全政银企社合作对接机制，搭建信息共享、资金对接平台，为企业发展和项目建设提供金融支持。持续深化商事制度改革，在“多证合一”改革基础上，积极探索“证照分离”改革、企业名称和简易注销登记改革，加快推进企业全程电子化登记改革，逐步推广电子营业执照，促进登记注册便利化。

积极推进农村“三变”改革试点。以农村“三变”改革为抓手，全面推进农村综合改革。深入推进农村土地集体所有权、农户承包权、土地经营权“三权分置”，健全完善农村产权交易市场体系，组建农村权益类资产评估机构，加快农村集体资产清产核资和确权工作。深化财政专项扶贫资金和其它涉农资金股权化改革，研究制定资源资金折股量化实施办法，明细资金种类、界定受益范围，规范程序和流程。通过多种模式培育壮大新型经营主体，以股权为纽带，建立健全农户与龙头企业、专业合作社、专业大户等更加紧密的利益联结机制。深入推进供销合作社综合改革，不断完善综合性、规模化、可持续的为农服务体系。

主动融入“一带一路”建设。依托全省推进国际产能和装备制造部省级合作平台，以有色、化工、能源等优势产业国际合作为先导，带动陶瓷建材、农特产品、中医药等特色产品进入国际市场。支持白银公司等骨干企业“走出去”，加大境外矿产资源合作勘探开发力度，进一步提高企业市场竞争力。加快白银公铁联运物流、鼎丰国际物流等陆港物流园区建设，提升白银至阿拉木图中欧货运班列运营能力。广泛开展项目洽谈、展会论坛以及各种经贸合作、文化交流等活动，进一步增强白银在区域协同发展中的地位和作用。

六、强化民生保障，推进共建共享，全面提升群众幸福指数。坚持以人民为中心的发展思想，多谋利民之事，多解民生之忧，进一步提升全市人民的获得感、幸福感和安全感。

着力完善社会保障体系。全力支持高校毕业生、农民工、复转军人、城镇失业人员等重点群体就业创业，城镇新增就业4.2万人以上，城镇登记失业率控制在4%以内。认真落实全民参保登记计划，扎实做好养老保险国家统筹政策衔接工作，确保全民参保登记率达到100%。上调失业保险金标准，逐步提高到最低工资标准的90%。健全完善社会救助、福利慈善、优抚安置等制度，切实保障困难群众基本生活。着力推进医养结合，继续开展养老院服务质量建设专项行动，提升养老服务质量和水平。大力发展残疾人事业，强化残疾康复服务。加强农村留守儿童和妇女、老年人关爱保护工作，健全完善关爱服务体系。

着力促进教育协调发展。加大城乡公办幼儿园建设力度，扩大公办学前教育资源覆盖面。推进义务教育均衡发展，着力解决义务教育发展不平衡不充分问题。实施普通高中质量提升和特色建设工程，集中力量改善高中阶段学校办学条件。加大职业教育资源整合，推动产教融合、校企合作，提高职业院校办学水平和教育质量。深化教师队伍管理体制改革，加强师资队伍建设，推进义务教育学校校长教师交流轮岗工作，优化县域城乡师资配置；落实乡村教师支持计划，进一步提高乡村教师待遇。

着力加强健康白银建设。全面落实卫生与健康事业优先发展实施意见，持续深化医药卫生体制改革，统筹推进公立医院综合改革、全民基本医保制度改革，加快医疗联合体建设，完善分级诊疗、家庭医生签约等制度，提升医疗综合服务能力。实施公共卫生服务能力提升行动计划，加快全科医生规范化临床培养基地建设。深入开展居民健康干预行动，全面落实重大疾病、慢性病防控措施，推进精神卫生、职业病防治服务体系和能力建设。加快景泰县中医院、平川中医院、会宁县人民医院业务综合楼等项目建设，改善医疗基础设施条件。加强基层医疗服务监管，规范医疗机构和医务人员诊疗行为，保障医疗安全。

着力发展文化体育事业。推进公共文化服务体系建设，全力做好全国第三批公共文化服务体系示范区创建迎接验收工作。推进县区“三馆”达标，完善乡镇（街道）综合文化站和村（社区）综合文化服务中心功能，推广地方数字文化资源建设。加强文物保护利用和文化遗产保护传承，实施好境内明长城、永泰城址、甘沟驿遗址、寺儿湾石窟等重点文物保护项目。广泛开展纪念改革开放40周年等系列文艺活动。落实全民健身计划，建成靖远县体育场、白银区全民健身中心，启动建设平川区体育中心。探索建立市场化体育赛事机制，提高竞技体育运动水平。

着力提升社会治理能力。健全多元化矛盾纠纷调解机制和社会稳定风险评估机制，深化基层治理、源头治理，有效化解社会矛盾。深化“七五”普法宣传教育，推动建立政府、社区、企业法律顾问制度。推进安全生产领域改革发展，落实“党政同责、一岗双责、齐抓共管、失职追责”安全生产责任制，加强安全生产信息化和应急救援能力建设，强化源头管控和安全准入，及时排查整治安全隐患，坚决遏制重特大事故发生。加强价格监管，稳定市场供应，维护消费者合法权益。健全守信激励和失信惩戒机制，规范信用体系建设。持续开展全省双拥模范城“九连冠”和全国双拥模范城“五连冠”创建活动，巩固和发展军政军民团结成果。加快推进社会治安防控体系建设，落实反恐防范措施，持续加强对社会治安、环境安全、食品药品安全等重点领域薄弱环节的隐患排查和整治，切实保障人民群众生命财产安全。

着力办好几件民生实事。（1）对全市60岁以上老年人实施意外伤害保险补助。（2）实施百名创客引领计划，对全市初始创业者给予5000元补助。（3）为全市95个社区综合文化中心配备1万元的文化活动设备。（4）投资6000万元，新建乡镇垃圾收集处理设施20个。（5）实现全市有需求的慢阻肺疾病筛查目标人群全覆盖。（6）积极推进“厕所革命”，新改建城乡公共厕所600座。（7）投资6000万元，改造提升城市公园广场8个。（8）建设“一村一场”农民体育健身广场100个。

各位代表！新时代要有新气象，更要有新作为。目标任务已经明确，关键在于狠抓落实。我们必须以改革创新精神加强政府自身建设，全面提升政府履职能力和水平，始终把抓落实作为政府工作的“生命线”，压实责任，主动担当，脚踏实地做好每一项工作，努力打造人民满意的服务型政府。

把牢政治方向，忠诚履职。坚持用习近平新时代中国特色社会主义思想武装头脑、指导实践、推动工作，切实增强“四个意识”，坚定“四个自信”，坚决维护以习近平同志为核心的党中央权威和集中统一领导。按要求认真开展“不忘初心，牢记使命”主题教育，持续推动“两学一做”学习教育常态化制度化。

严格依法行政，规范用权。全面加强法治政府建设，提高运用法治思维和方式解决问题的能力。依法履行政府职能，积极办理人大代表建议和政协提案，严格执行市政府工作规则和重大行政决策法定程序，落实行政执法责任制。深入推进政务公开，完善行政权力制约和监督机制，主动接受监督，做到依规依纪依法用权。

强化责任担当，狠抓落实。大力弘扬真抓实干精神，大兴调查研究之风，及时细化分解任务，真正沉下心来、扑下身子，以“钉钉子”精神抓好落实，确保件件有着落、事事有回音。加大政务督查督办力度，运用好、转化好督查成果。完善绩效管理和考核评价体系，探索建立重大政策落实第三方评估制度。健全容错纠错激励机制，旗帜鲜明为敢于担当、创新探索、踏实做事、不谋私利的干部撑腰鼓劲。完善问责和激励机制，推进追责问责制度化、常态化，褒奖埋头苦干、狠抓落实的干部，以奖惩分明促勤政有为。

坚持廉洁从政，干净干事。切实履行全面从严治党主体责任和党风廉政建设“一岗双责”，严格执行中央八项规定实施细则和省市委相关规定，持之以恒纠正“四风”。加强审计监督，严控行政支出，用制度管住权力、预防腐败，严肃查处各类违纪违法案件，努力促进干部清正、政府清廉、政治清明。

各位代表！奋斗成就梦想，实干赢得未来。让我们更加紧密地团结在以习近平同志为核心的党中央周围，坚定不移贯彻中央和省委、省政府各项决策部署，在市委的坚强领导下，不忘初心，牢记使命，振奋精神，开拓进取，为建设繁荣富裕、文明和谐、美丽宜居的现代化新白银而不懈奋斗。
